# Preparation and physicochemical characterization of camptothecin conjugated poly amino ester–methyl ether poly ethylene glycol copolymer

**DOI:** 10.1039/c8ra01407h

**Published:** 2018-04-06

**Authors:** Ali Fattahi, Nadia Karimi, Fatemeh Rahmati, Yalda Shokoohinia, Komail Sadrjavadi

**Affiliations:** Medical Biology Research Center, Kermanshah University of Medical Sciences Kermanshah Iran; Department of Chemistry, Basic of Sciences Faculty, Ilam University Ilam Iran; Student Research Committee, School of Pharmacy, Kermanshah University of Medical Sciences Kermanshah 6734667149 Iran; Pharmaceutical Sciences Research Center, School of Pharmacy, Kermanshah University of Medical Sciences Kermanshah 6734667149 Iran komail.sadrjavadi@gmail.com

## Abstract

In the present study, camptothecin grafted poly amino ester-methyl ether polyethylene glycol (CPT-PEA-MPEG) as a novel copolymer was synthesized by Michael reaction at different ratios of MPEG and CPT (60 : 40 and 80 : 20). The microemulsion was used to prepare nanomicelles, and *in vitro* cytotoxicity was performed on the HT29 cell line, and cell survival was measured by MTT assay. The syntheses were confirmed by ^1^H NMR and FT-IR. Several characterization methods including CMC, particle size, size distribution, and transmission electron microscopy were performed to evaluate features of prepared nanomicelles. Low critical micelle concentration, small particle size and IC_50_ of 0.1 mg ml^−1^ at MPEG to CPT ratio of 60 : 40 make this micelle a promising drug delivery carrier. CPT-PAE-MPEG nanomicelles at a MPEG : CPT ratio of 60 : 40 can be a suitable choice to improve the physiochemical properties of CPT and its therapeutic effect, while it can be potentially used as a nano-carrier for other anticancer drugs to purpose a dual drug delivery.

## Abstract 2

In the present study, camptothecin grafted poly amino ester-methyl ether polyethylene glycol (CPT-PEA-MPEG) as a novel copolymer was synthesized by Michael reaction at different ratios of MPEG and CPT (60 : 40 and 80 : 20).

## Graphical abstract

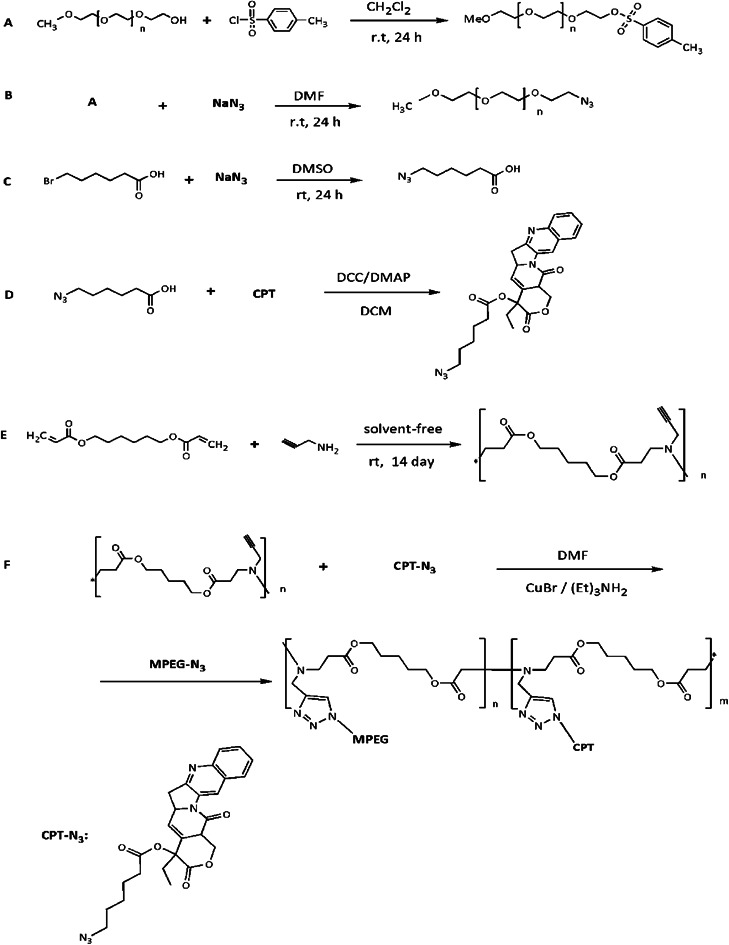

## Introduction

Cancer is one of the most devastating diseases causing huge mortality around the world. Current cancer therapy suffers from some vital drawbacks such as non-specific targeting, drug resistance, poor bioavailability, low solubility in water, and also toxicity for normal cells resulting in patient toxicity. Nano drug delivery systems, *e.g.*, polymeric nanoparticles, nanomicelles, and polymersomes are promising candidates to overcome such drawbacks and provide selective drug delivery which possesses the ability to be designed for optimal size and surface characteristics.^1,2^

Polymeric micelles introduced as drug delivery vehicles in the 1980s by Helmut Ringsdorf and co-workers^3^ have manifested several advantages over other types of carrier. Their core–shell structure with the drug in the core guarantees low toxicity in the human body, and their prolonged circulation time in the bloodstream is owing to the high hydrophilic shell which prevents phagocytic and renal clearance.^4^ Furthermore, they possess proper stability, bioavailability and strong mechanical properties.^5^

Recently, co-delivery of small molecule anticancer drugs, macromolecular genes and therapeutic proteins has attracted more attention.^6,7^ Accordingly, different types of polymeric micelles were used for co-delivery of drugs and genes.^8–10^ Among dual drug delivery systems, drug conjugated polymeric micelles were more interesting because the hydrophobic drugs play two main roles simultaneously as hydrophobic apart of micelles and efficient drug.^11–13^

Poly amino ester is a biodegradable and biocompatible cationic polymer easily synthesized from primary amines and diacrylate esters.^14,15^ It has the high buffer capacity due to its tertiary amine groups^16^ and has been used excessively in drug and gene delivery.^17^

Polyethylene glycol (PEG) is one of the most suitable polymers used for biotechnological and biomedical applications. Due to the high hydrophilic properties, solubility in aqueous and organic solvents and absence of antigenicity and immunogenicity, it is utilized for hydrophilic outer shell in preparation of polymeric micelles.^18^ Although adding PEG or any other hydrophilic polymer into the structure of the nano-particular system can reduce cell uptake, this disadvantage can be justified by using specific ligands, *e.g.*, monoclonal antibodies and aptamers. Camptothecin is a five-ring alkaloid and a topoisomerase inhibitor, effective on a wide range of tumors. It has a limited therapeutic application due to its instability at neutral pH, low solubility in aqueous medium and nephrotoxicity. Polymeric micelles are among novel pharmaceutical carriers widely used to minimize or eliminate undesired physiochemical characteristics of CPT.^19^

The aim of the present study is the synthesis of a new generation of polymeric micelles with inherent anticancer activity by synthesis of CPT-PAE-MPEG copolymer and preparation of its micelles.

## Materials and methods

### Materials

1,6-hexanediol diacrylate, 6-bromohexanoic acid, 4-toluenesulfonyl chloride (Tos-Cl), propargylamine, sodium azide (NaN_3_), *N*,*N*′-dicyclohexylcarbodiimide (DCC) and 4-dimethyl aminopyridine (DMAP) were purchased from Sigma [St. Louis, MO, USA]. Methoxy poly (ethylene glycol) [MPEG; number-average molecular weight (*M*_n_) = 2000, Sigma-Aldrich] was dried over anhydrous toluene by azeotropic distillation. Dimethylsulfoxide (DMSO), diethyl ether, methanol (MeOH), chloroform (CCl_3_), dimethylformamide (DMF), tetrahydrofuran (THF), ethyl acetate (EtOAc), toluene, and acetone [Merck A.G., Germany] included in the polymerization recipe as a diluent were used without further purification. Dichloromethane (DCM) was obtained from Merck and dried by refluxing over calcium hydride at 60 °C for two h, and it was distilled immediately before use. Mineral materials, *e.g.*, CuBr, CaCl_2_, NaCl, and NaHCO_4_, were purchased from Merck, Germany.

### Methods

#### Synthesis of MPEG-N_3_

##### Synthesis of MPEG-Tos

**Fig. 1:**
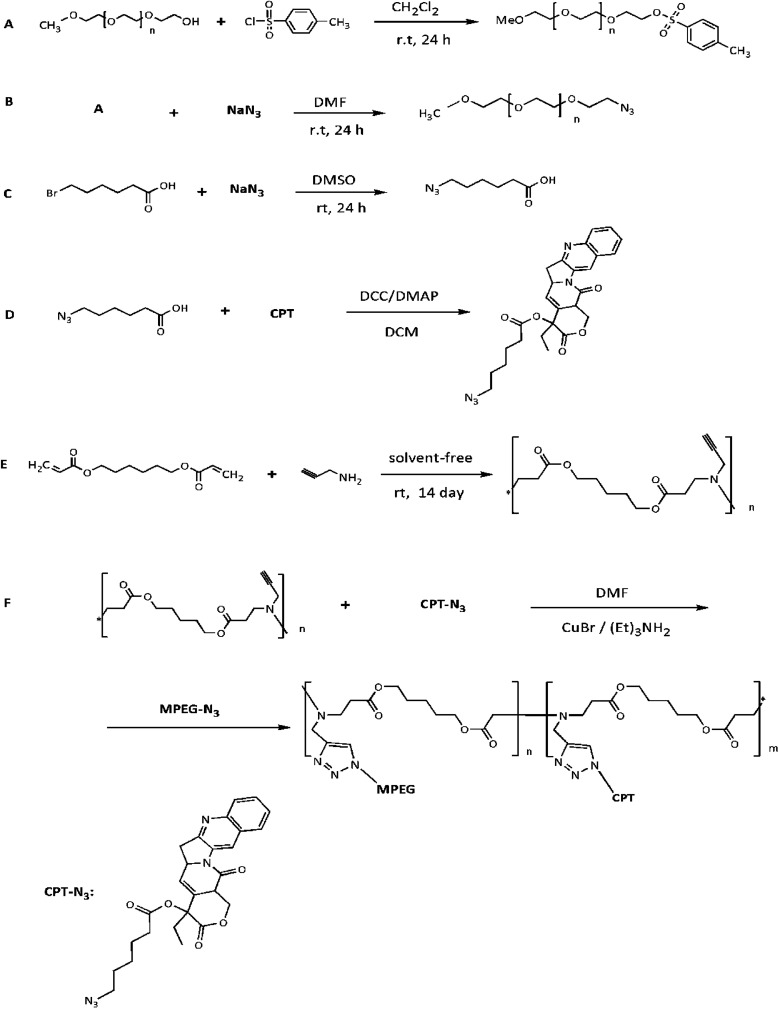
Schematic of synthesis of MPEG-Tos (A), MPEG-N_3_ (B), 6-azidohexanoic acid (C), camptothecin-azide (D), poly amino ester (E) and CPT-PAE-MPEG copolymer (F).

MPEG-N_3_ was synthesized in two steps; at first, MPEG (2 g, 1 mmol) was dissolved in 15 ml of DCM and triethylamine (2.78 ml, 20 mmol). Then DMAP (1.22 g, 10 mmol) and Tos-Cl (1.91 g, 10 mmol) were added to the mixture. The reaction mixture was stirred for 24 h at room temperature under a nitrogen atmosphere (Fig. 1A). Finally, hydrochloric acid (4 M) was added, and the reaction mixture was washed thrice with 100 ml of acid, and the residue was dried using anhydrous CaCl_2_, followed by filtration to remove the salt. The evaporation of the solvent was carried out in vacuum. The resulting product was purified using column chromatography over silica gel; at first, with CH_2_Cl_2_/EtOAc mixture (ratio of 1 : 1) and then with MeOH/CH_2_Cl_2_ mixture (ratio of 1 : 10). The resulting product was dissolved in tetrahydrofuran (THF), precipitated in cold diethyl ether, and dried in a vacuum oven (85% yield).^20^ The synthesis was proved using FT-IR.

FT-IR (KBr): *ν*_max_ (cm^−1^) = 3069 (=C–H), 2935 and 2885 (–C–H), 1597 and 1465 (C=C, ring), 1346 (S=O), 1141 and 1114 (C–O), 842 and 775 (S–O).

##### Synthesis of MPEG-N_3_

MPEG-Tos (500 mg, 0.25 mmol) was dissolved in minimum DMF (less than 15 ml), and then NaN_3_ (6.6 mg, 0.1 mmol) was added. The reaction mixture was stirred for 24 h at room temperature under the nitrogen atmosphere (Fig. 1B). After evaporation of the solvent in vacuum, the precipitate was dissolved in DCM (100 ml). The solution was washed thrice with NaCl (5%) and distilled water. The solvent was evaporated in vacuum, and it was precipitated in cold diethyl ether. The product was dried in a vacuum oven (85% yield). Structure of MPEG-N_3_ was proved by FT-IR.^21^

FT-IR (KBr): *ν*_max_ (cm^−1^) = 2935 and 2885 (–C–H), 2100 (–N_3_), 1465 (C–N), 1141 and 1114 (C–O).

#### Synthesis of camptothecin-azide

CPT-N_3_ was synthesized in two steps.

##### Synthesis of 6-azidohexanoic acid

6-bromohexanoic acid (500 mg, 2.56 mmol) and NaN_3_ (420 mg, 6.4 mmol) were added to a reaction flask and dissolved in DMSO (20 ml). The reaction mixture was stirred for 24 h at room temperature under nitrogen atmosphere. Then, the reaction mixture was washed with CH_2_Cl_2_ (30 ml), and the organic layer was separated and washed three times with NaCl (26%) and distilled water. Again, the resulting mixture was washed with saturated aqueous NaHCO_3_ and dried with anhydrous CaCl_2_. The final 6-azidohexanoic acid was obtained (25% yield) after removing the solvent. The reaction is shown in Fig. 1C, and structure of the product was proved by FT-IR and ^1^H NMR spectroscopy.

FT-IR (KBr): *ν*_max_ (cm^−1^) = 2500–3400 (OH), 2959 and 2868 (–C–H), 2096 (–N_3_), 1708 (C=O) 1458 (C–N), 1257 (C–O).

^1^H NMR (400 MHz; DMSO-d_6_): *δ* = 1.32 (t, 2H), 1.49, 2.21 (2m, 4H, 2CH_2_), 2.54 (t, 2H, CH_2_C=O), 3.32 (t, 2H, CH_2_–N_3_), 11.88 (brs, 1H, OH).

##### Synthesis of camptothecin-azide

The CPT-N_3_ was synthesized by the reaction between CPT (100 mg, 0.27 mmol) with azidohexanoic acid (85 mg, 0.54 mmol) (4 mg, 0.03 mmol) in dry CH_2_Cl_2_ and at the presence of DMAP/DCC.^22^ CPT, azidohexanoic acid, DMAP, and CH_2_Cl_2_ (5 ml) were added to a reaction bottle. The reaction mixture was cooled down to 0 °C, and DCC (110 mg, 0.54 mmol) was added. The reaction mixture was stirred for 2 h under nitrogen atmosphere. Then the mixture reaction was stirred at room temperature for 6 h. The product was precipitated, and it was crystallized from a Me–OH/DCM mixture (with a ratio of 95 : 5). For this purpose, the reaction mixture was kept at a temperature in the range of 30–40 °C for 30 min until a clear solution was obtained. Then, the solution was kept at a temperature of 0 °C overnight until CPT-N_3_ precipitate was obtained. The product was dissolved in CHCl_3_ and was centrifuged at 8800 rpm for 20 min. The solution was separated and was dried in vacuum (45% yield).^23^ Structure of CPT-N_3_ was proved by FT-IR and ^1^H NMR data. The reaction is shown in Fig. 1D.

FT-IR (KBr): *ν*_max_ (cm^−1^) = 3082 (=C–H, ring), 2939 and 2870 (–C–H), 2100 (–N_3_), 1728 (C=O of ester), 1747 (C=O of lactone), 1666 (C=O of amide) 1562 and 1454 (C=C ring), 1624 (C=N), 1346 (C–N), 1230 and 1149 (C–O, ring), 1118 (C–O).

^1^H NMR (400 MHz; DMSO-d_6_): *δ* = 0.99 (t, CH_3_, aliphatic), 1.91 (t, 2H, CH_2_), 1.38–1.61, (m, CH_2_, aliphatic), 2.33 (t, 2H, CH_2_C=O), 2.5 (t, 2H, CH_2_–N_3_), 2.96, 3.17 (s, 2H, CH_2_–N, ring), 4.29 (s, 2H, CH_2_–O, ring), 5.76–7.72 (m, 6H, CH of ring).

#### Synthesis of poly amino ester

PAE was synthesized *via* Michael addition reaction.^24^ 1,6-Hexanediol diacrylate (1.15 ml, 3.23 mmol) and propargylamine (0.416 ml, 6.47 mmol) were added into a brown reaction vial, far from the light, under solvent-free condition (Fig. 1E). The mixture was stirred for 7 days at room temperature under nitrogen atmosphere. After 7 days, an excess of propargylamine (0.04 ml, 0.6 mmol) was added to consume any unreacted 1,6-hexanediol, and stirring was continued for further 7 days at 25 °C. The product was precipitated in cold diethyl ether. The solid residue was separated and was dried with vacuum to obtain the product (with a yield of 95%). Structure of PAE was proved by FT-IR and ^1^H NMR data.

FT-IR (KBr): *ν*_max_ (cm^−1^) = 3444(–N–H), 3278 (≡C–H), 2927 and 2857 (–C–H), 2102 (–C≡C), 1728 (C=O of ester), 1462(–N–H), 1327 (C–N), 1257 (C–O), 732 (–N–H, oop).

^1^H NMR (400 MHz; DMSO-d_6_): *δ* = 1.27, 1.39, 1.64 (m, 12H, CH_2_, aliphatic), 2.22 (s, 1H, –C≡C–H), 2.54 (t, 2H, CH_2_C=O), 2.85 (t, 2H, CH_2_–N), 3.44 (s, 2H, N–CH_2_–), 4.09 (t, 4H, CH_2_–O).

#### Synthesis of CPT-PAE-MPEG

CPT-PAE-MPEG copolymer was prepared *via* click chemistry. Click chemistry is the joining reaction between an alkyne and N_3_ in the presence of copper catalysis utilizing 1.3-dipolar cycloaddition.

CPT-PAE-MPEG was synthesized using a mixture of MPEG-N_3_ and CPT-N_3_ at two ratios of MPEG : CPT (60 : 40 and 80 : 20).

To synthesize CPT-PAE-MPEG copolymer at a ratio of 80 : 20, PAE (19.7 mg, 0.06 mmol) and CPT-N_3_ (5.58 mg, 0.012 mmol) were dissolved in 5 ml anhydrous DMF. Then CuBr (3.5 mg, 0.024 mmol) as the catalyst and triethylamine were added to the mixture (1.08 mg, 0.024 mmol). This mixture was stirred at room temperature for 48 h under nitrogen atmosphere. Then, MPEG-N_3_ (98.68 mg, 0.048 mmol) was added, and the reaction was stirred under the condition described above. After 48 h, the excess amount of MPEG-N_3_ (115 mg, 0.055 mmol) was added to consume unreacted acetylene group of PAE, and it was stirred for further 48 h. Finally, the reaction mixture was centrifuged at 7000 rpm for 20 min. The solution was separated and precipitated with cold diethyl ether, and it was dried with the evaporation of the solvent to obtain viscous yellow oil. The finished product was purified by dialysis (cut-off 3500 Da) with buffer solution (pH 7.4) for 48 h at 4 °C. The final CPT-PAE-MPEG copolymer was synthesized with yield of 60%, and the structure of copolymer was proved by FT-IR and ^1^H NMR data. The reaction is shown in Fig. 1F.

To synthesize CPT-PAE-MPEG copolymer with ratio of 60 : 40, PAE (9.85 mg, 0.03 mmol), CPT-N_3_ (11.16 mg, 0.024 mmol) CuBr (1.75 mg, 0.012 mmol) and MPEG-N_3_ (76.14 mg, 0.036 mmol) were added to DMF (5 ml). The reaction was allowed to continue in different steps similar to the reaction of 80 : 20. The final CPT-PAE-MPEG copolymer was synthesized with yield of 50%.

FT-IR (KBr): *ν*_max_ (cm^−1^) = 3458 (–N–H), 3007 (=C–H, ring), 2927 and 2857 (–C–H), 1732 (C=O, ester of PAE and CPT), 1748 (C=O of lactone of CPT), 1656 (C=O of amide of CPT) 1562 and 1452 (C=C ring, of CPT), 1624 (C=N of CPT), 1357 (C–N of CPT and PAE), 1230 and 1149 (C–O, ring of CPT), 1118 (C–O).

^1^H NMR (400 MHz; DMSO-d_6_): *δ* = 1.17, 1.26, 1.39 (m, 12H, CH_2_, of PAE chine), 2.33 (t, 2H, CH_2_C=O, of PAE), 2.56 (t, 2H, CH_2_–N, of polymer), 0.95 and 1.7 (m, 4H, CH_2_, of CPT-N_3_ chine) 2.95 (t, 2H, CH_2_–N_3_, of CPT-N_3_ chine), 2.31 (t, 2H, CH_2_–C=O, of CPT-N_3_ chine), 3.44 (s, 2H, –N–CH_2_–teryazol ring), 3.82 (t, 4H, –O–CH_2_–CH_2_–O–, of PEG-N_3_) 4.09 (t, 4H, CH_2_–O, of PAE), 7.03 (1H, CH, of teryazol ring), 6.1–7.80 (m, 6H, CH, of CPT-N_3_ ring).

### Copolymer characterization

#### FT-IR (fourier transform infrared spectroscopy)

FT-IR spectra of compounds were obtained with FT-IR spectroscopy (IR prestige-21, Shimadzu Co., Japan) in the spectral range of 400–4000 cm^−1^ at a resolution of 4 cm^−1^ and room temperature using KBr disc.

#### ^1^H NMR

^1^H NMR spectra were obtained on a Broker MSI-400 at 400 MHz. Chemical shifts are in part per million (ppm) downfield from TMS as an internal standard, and samples were dissolved in DMSO-d_6_ for analysis.

#### Critical micelle concentrations of CPT-PEA-MPEG copolymer

The critical micelle concentration (CMC) was measured with the fluorescent method and using Nile Red as a fluorescent indicator with a concentration of 2.5 × 10^−8^ M. For the CMC study, CPT-PEA-MPEG micelles at MPEG : CPT ratio of 60 : 40 and 80 : 20 were prepared with serial dilution (0.25–2.47 × 10^−7^ and 0.05–1.95 × 10^−5^ mg ml^−1^, respectively) using distilled water. The obtained solutions were sonicated for 30 min, and then the emission spectra of Nile Red were recorded at 512 nm (the exciting wavelength was 482 nm, and the emission wavelength range were 500–650 nm). The fluorescent intensity was measured using a fluorescence spectrophotometer (Perkin-Elmer, LS-45, U.K).

#### Molecular weight determination

The molecular weight of the polymer was measured by static light scattering (SLS) technique using Zetasizer (Nano-ZS, Malvern Instruments Ltd., Worcestershire, UK). For this step, six different concentrations of solutions (0.01–0.06 mg ml^−1^) were prepared, and molecular weights were recorded using the Malvern supplied molecular weight operating procedure. Toluene (the solvent for polymer) was used as the reference.

#### Micelles characterization

##### Preparation of nanomicelles

The nanomicelles were prepared by microemulsion technique. 1.25 mg of the synthesized copolymer was added to 2 ml of DCM at room temperature. Then, the organic phase was added to 5 ml of DI water (aqueous phase) under rapid stirring condition.

The obtained emulsion was sonicated with a 20 kHz sonicator (GM 2070, Bandelin, Germany) for 2 min at 70% power, and then it was stirred at room temperature to evaporate DCM.

##### Measurement of particle size

The mean particle size (*Z*-average) of nanomicelles was measured using Zeta seizer (ZS300, Malvern Co., UK) at room temperature. For this step, the solution was prepared, and it was filtered with 400 nm filter to remove any dust and impurity. The average size of the nanomicelles was determined in two different MPEG : CPT ratios (80 : 20 and 60 : 40).

##### Transmission electron microscope

The morphology of nanomicelles was studied using a Transmission Electron Microscope (TEM, Zeiss – EM10C −80 kV, Germany). For the preparation of the sample, a drop of micelles at the ratio of 60 : 40 (0.25 μg ml^−1^) was placed on a carbon-coated copper grid and then dried under air atmosphere at room temperature.

##### Yield of CPT conjugation

The amount of conjugated CPT in CPT-PEA-MPEG micelles was determined using UV spectrophotometer (Shimadzu, 1240 UV-Vis., Japan). Briefly, 1 mg of CPT-PEA-MPEG micelles at MPEG : CPT ratio of 60 : 40 was dissolved in 600 μl of EtOH, and then 400 μl of distilled water was added to the solution. The absorption of the diluted solution was determined by UV spectrophotometer at 370 nm. The calibration curve was prepared for the quantification of CPT at the CPT concentrations of 439, 219, 109, 54 and 27 μg ml^−1^ with a correlation coefficient of *R*^2^ = 0.9980. The yield of CPT conjugation was obtained as the mass ratio between the conjugated amount of CPT in micelles and the initial amount of drug used in the preparation.

##### Release study

The *in vitro* release of CPT-PEA-MPEG micelles with MPEG : CPT ratio of 60 : 40 was measured in PBS (phosphate buffer solution, 0.01 N) containing EtOH (3%, v/v) at 37 °C. Four mg of micelles was suspended in 4 ml of PBS at PH 7.4 or 5.8. Then, the micelles solution was placed into a dialysis bag (molecular weight cut-off 8000), and the dialysis bag was plunged in 40 ml of PBS containing 3% v/v EtOH and 0.1% w/v NaN_3_ (to prevent bacteria and fungi growth) at pH 7.4 or 5.8. The temperature was kept constant at 37 °C with horizontal shaking at 120 rpm in a shaker (NB-205, N-Biotek, Korea) for 11 days. At the designated time intervals, 2 ml of release medium was withdrawn, and the amount of drug release for samples was assessed by UV-Vis spectrophotometer at 370 nm. The withdrawn medium was replenished with fresh release medium.^25^

##### *In vitro* cytotoxicity tests

*In vitro* cytotoxicity test was performed using HT29 cell line were obtained from Pasteur Institute (Tehran, Iran). The cells were cultured in RPMI-1640 containing 10% FBS and penicillin-G/streptomycin solution (100 mg ml^−1^ – 100 U ml^−1^). These cells were placed in an incubator under conditions of 95% moisture, 5% CO_2_, and temperature of 37.5 °C for 24 h. Nanomicelles with concentrations in the range of 0.0062–0.1 mg ml^−1^ were added to microplates. The cell survival was measured using 3-(4,5-dimethylthiazol-2-yl)-2,5-diphenyl tetrazolium bromide (MTT) test. After 48 h, 20 μl of MTT solution (0.1 mg ml^−1^) was added to microplates, and the cells were incubated for 3 h. Then, the content of wells was dissolved in DMSO (150 μl per well). Formazan product was detected by measuring absorbance with an ELISA plate reader (BioTek, H1M, USA) at a test wavelength of 540 nm and a reference wavelength of 630 nm to obtain sample signal as the following formulation:

1 Cell viability (%) = (OD540_s_ − OD630_s_)/(OD540_c_ − OD630_c_) × 100

where s is sample and c is control.

## Results and discussion

### Synthesis and characterization of polymers

MPEG-Tos, MPEG-N_3_, CPT-N_3_, PAE and CPT-PAE-MPEG copolymers were synthesized, and their structures were confirmed by FT-IR and ^1^H NMR. The yield and changing the color of products were reported in each part. The characterization of each product was studied separately.

### FT-IR spectroscopy analysis

The FT-IR spectrum of the prepared PEG-Tos indicated that the characteristic peak at 1176 and 1346 cm^−1^ were related to symmetrical and asymmetrical stretching vibrations of S=O. The C=C stretching vibrations of the aromatic ring was observed at 1597 and 1465 cm^−1^, and the peak at 3057 cm^−1^ was related to the C–H stretching vibration of the aromatic ring. Also, the broad peak of OH group of PEG at 3417 cm^−1^ was disappeared. These results confirmed the presence of tosyl group. The spectrum of PEG-N_3_ revealed the characteristic peak of N_3_ stretching vibrations at 2100 cm^−1^ in the PEG-N_3_, and the omitting of PEG-Tos peaks at 1570 and 1176 cm^−1^ (relating to benzyl ring and S=O, respectively) confirmed the presence of N_3_.

PAE was synthesized by two monomers; propargylamine and 1,6-hexanediol diacrylate. The spectrum of propargylamine showed the characteristic peaks of N–H primary amine stretching vibration and bending vibration of the propargyl at 3429, 3600 and 1635 cm^−1^. Also, the sharp peak located at 2150 cm^−1^ identified the acetylene stretching vibration, and the peak at 3290 cm^−1^ indicated the symmetrical C–H stretching of acetylene group of the propargyl. The 1,6-hexanediol diacrylate spectrum showed the characteristic of C–H alkenes stretching vibration peak at 3105 cm^−1^, and the peak at 1635 cm^−1^ indicated the C=C alkenes stretching vibration. The synthesized PAE showed the C–H alkenes stretching vibration peak at 3287 cm^−1^, and the sharp peak at 2102 cm^−1^ identified C≡C stretching in propargylamine. Also, the peak at 3444 cm^−1^ was characteristic of N–H stretching vibration of the second amine, and the peak at 3051 cm^−1^ could be related to C–H alkene stretching vibration. The characteristic peak of C–H alkenes stretching vibration at 3105 cm^−1^ and the peak of C=C alkenes stretching vibration at 1635 cm^−1^ were omitted in the polymer spectrum, which confirmed the reaction between 1,6-hexanediol diacrylate and propargylamine.

The esterification between OH group of CPT and the carboxylic acid group of 6-azidohexanoic acid resulted in CPT-N_3_. The spectrum of 6-azidohexanoic acid showed the broad peak of OH stretching vibration in the range of 2400–3600 cm^−1^, and the sharp peak of C=O stretching vibration of carboxylic acid at 1708 cm^−1^. Also, this spectrum identified a sharp peak of – the N_3_ stretching vibration of 6-azidohexanoic acid at 2100 cm^−1^, and the spectrum of CPT showed a broad peak of OH stretching vibration at 3433 cm^−1^ while the spectrum of the CPT-N_3_ indicated a peak observed at 1747 cm^−1^ region for the ester carbonyl stretching vibration. The presence of this peak and disappearing peak at 3433 cm^−1^ confirmed the esterification and the presence of CPT-N_3_.

We applied the click chemistry between acetylene group of PAE and N_3_ group of CPT-N_3_ and MPEG-N_3_ to synthesize CPT-PAE-MPEG. The FT-IR spectrum of CPT-PAE-MPEG were indicated the peaks of the CH_2_ and CH_3_ symmetric, and asymmetric stretching vibrations from 2854 to 2927 cm^−1^ and a weak vinyl C–H stretching vibration peak appeared at 3007 cm^−1^. The carbonyl bands from ester group of PAE, lactone and amide group of CPT were indicated at 1732, 1748 and 1656 cm^−1^, respectively. The peaks at 1562 and 1452 cm^−1^ were attributed to the symmetrical C=C stretching of aromatic ring group of CPT, and the peak at 1624 cm^−1^ was corresponded to the C=N stretching vibration of CPT, although the peak at 1624 cm^−1^ was overlapped by the peak of C=C vinyl stretching vibration of triazole ring. The peak at 1118 cm^−1^ was corresponded to –C–O– of MPEG. Furthermore, the peaks of C≡C (at 2150 cm^−1^ region) and ≡C–H (3290 cm^−1^ region) related to PAE and peak of N_3_ (at 2100 cm^−1^ region) corresponding to PEG-N_3_ and CPT-N_3_ were disappeared in CPT-PAE-MPEG spectrum synthesis of the copolymer.

### ^1^H NMR spectroscopy analysis

^1^H NMR data for 6-bromohexanoic acid indicated the peaks at 3.5, 2.21 and 11.89 ppm, which were respectively attributed to the protons of the –CH_2_Br, –CH_2_COOH and –COOH. The spectrum of 6-azidohexanoic acid revealed the peaks at 3.32, 2.54 and 11.88 ppm that were respectively related to protons of the –CH_2_N_3_, –CH_2_COO and –COOH. These data confirmed the successful synthesis of 6-azidohexanoic acid. Then, the carboxylic acid group of 6-azidohexanoic acid was grafted into hydroxyl group of CPT. The characteristic peaks of CPT-N_3_ at 2.33 and 2.5 ppm identified protons of the –CH_2_COO-CPT and –CH_2_N_3_ groups of 6-azidohexanoic, and the peak at 11.88 ppm related to the proton of –COOH group was omitted. These peaks confirmed the conjugation of 6-azidohexanoic acid to CPT. Also, the peaks at 5.76–8.15 ppm assigned to the phenyl ring and protons of the double bend of CPT confirmed the presence of CPT in the structure.^26^ In the spectrum of MPEG, the identified a peck at 3.4 ppm indicated the protons of ethylene group.

PAE showed the peak of –CH_2_NH– at 3.44 ppm and the peak of methylene of propargylamine group at 3.69 ppm. The peak at 2.47 indicated protons of –CH_2_COO– and the peak at 4.08 identified protons of –CH_2_OOC– group. Also, the peak at 2.85 ppm was characteristic of proton of propargylamine group (–C≡CH), the peak relating to the proton alkenes of 1, 6-hexanediol diacrylate group at around 6 ppm was omitted. These results confirmed the synthesis of PAE from 1,6-hexanediol diacrylate and propargylamine. In the spectrum of CPT-PAE-MPEG, the peaks relating to protons of –O–CH_2_–CH_2_–O and –OCH_3_ of MPEG unit at 3.82 and 4.09 ppm, were observed, respectively. The peaks relating to protons of C–H aromatic ring of CPT at 6.1–7.80 ppm were observed, and also the peak relating to the proton of C–H double bond triazole ring at 7.03 ppm was observed. The results of ^1^H NMR spectra confirmed the synthesis of CPT-PAE-MPEG.

### Molecular weight analysis

The molecular weight of PAE was evaluated with the Zetasizer Nano system. The molecular weight was obtained about 11.1 kD which is similar to results obtained in other studies. The derivative of PAE containing secondary amine group had a molecular weight in around 6 to 10 kD.^27^

#### Characterization of micelles

##### Particle size analysis and stability of nanomicelles

The particle sizes and PDI were analyzed for CPT-PAE-MPEG nanomicelles with different ratios of MPEG to CPT (60 : 40 and 80 : 20). The mean particle size of nanomicelles at ratios of 60 : 40 and 80 : 20 was 118.9 ± 1.66 and 166.6 ± 26.79 nm, and their PDIs were 0.108 ± 0.014 and 0.292 ± 0.018, respectively. The size and PDI of nanomicelles decreased with increasing the percentage of conjugated CPT. Increasing the degree of substitution increases the hydrophobicity and consequently forming a denser core, which could be the reason for decreasing the size of 60 : 40.^28^ The reduced trend of size in the hydro state in this study agrees with a similar study carried out by Yang *et al.*;^29^ the higher percentage of the drug is, the higher hydrophobic interaction at the core of micelles is.

**Fig. 2:**
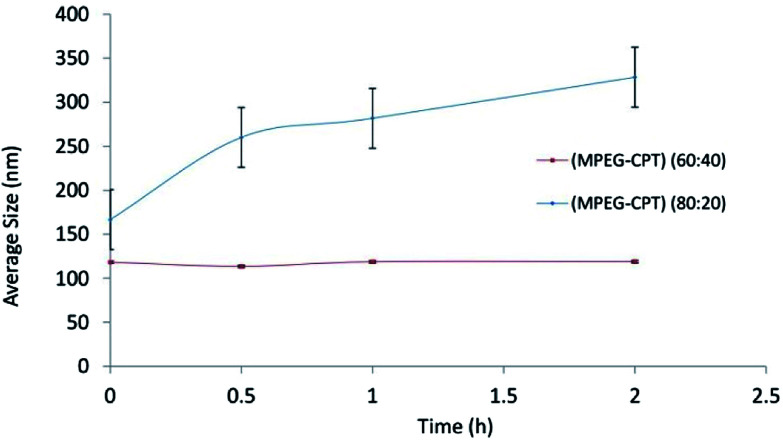
The size stability of nanomicelles at ratios of 60 : 40 and 80 : 20.

The results of stability of nanomicelles for ratios of 60 : 40 and 80 : 20 are shown in Fig. 2. At the ratio of 80 : 20, not only particle size showed enhancement during the time, but also standard deviation (S.D.) was significantly higher than that one at ratio 60 : 40. The lower S.D. is, the higher stability is. According to reasons above, the stability of 60 : 40 could be related to the formation of the denser core and lower CMC value.^28^

##### Critical micelle concentrations of CPT-PAE-MPEG copolymer

**Fig. 3:**
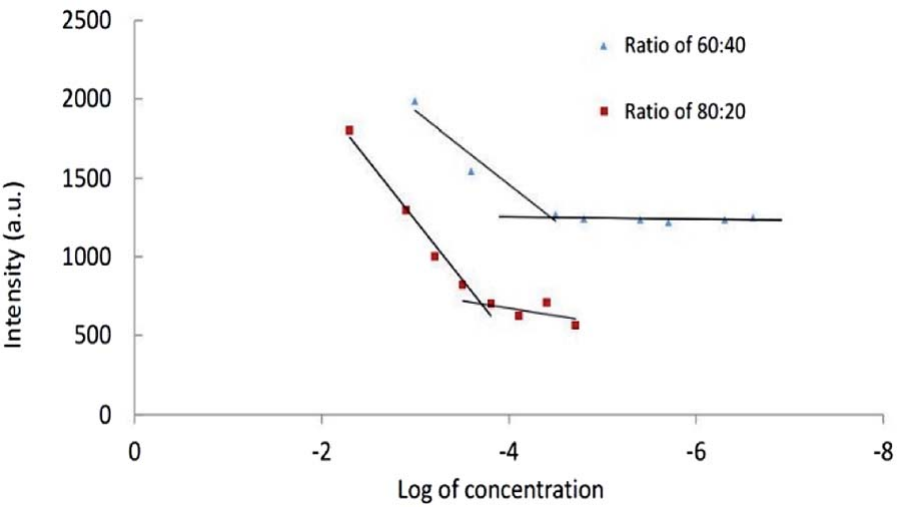
The critical micelle concentrations of nanomicelles at different ratios of MPEG : CPT (60 : 40 and 80 : 20).

The result of critical micelle concentrations (using Nile Red) is shown in Fig. 3. The critical micelle concentration (CMC) was 3.55 × 10^−5^ mg ml^−1^ at ratio of 60 : 40 and 1.96 × 10^−4^ mg ml^−1^ at ratio of 80 : 20.

Low CMC is one of the most important characters of nanomicelles for *in vivo* applications. Nanomicelles with lower CMC have enhanced stability in body fluids. The drug-loaded polymeric micelles with high CMC led to an infinite dilution in the bloodstream, which may cause the dissociation of the polymeric micelle and premature drug release. The inherent CMC values of polymeric micelles are influenced by the molecular weight of block copolymers, the hydrophobicity of hydrophobic block, as well as the block length and ratio of blocks.^30^ Increasing the CPT percentage leads to an increase in hydrophobic interaction at the core of micelles and causes facilitating the formation of nanomicelles. Inter-molecular interactions such as electrostatic interactions, chemical cross-linking, and hydrogen bonding can enhance stability.^31^

##### Morphology of nanomicelles

**Fig. 4:**
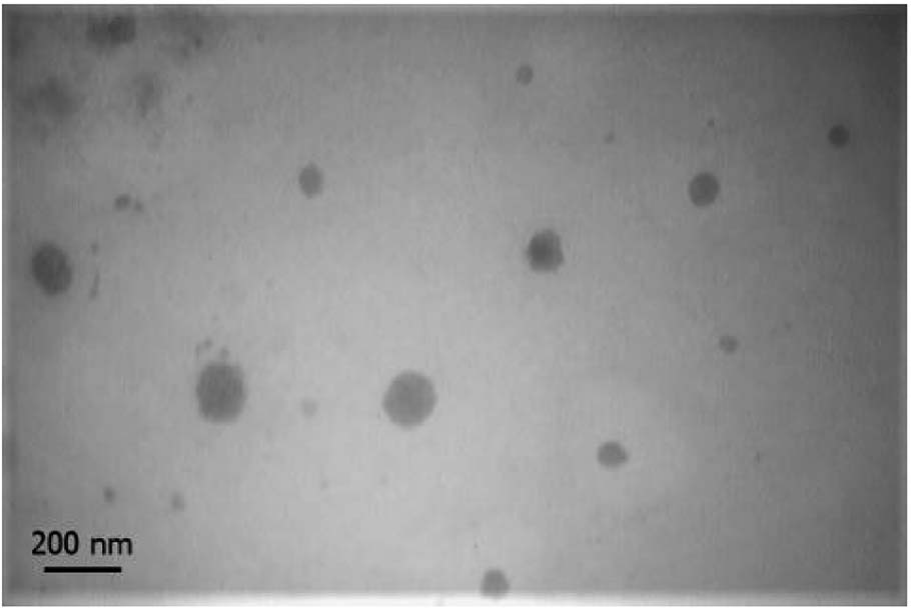
TEM image of nanomicelles at ratio of 60 : 40 in 20000× magnification.

Transmission electron microscope image is shown in Fig. 4. Nanomicelles at the ratio of 60 : 40 have a spherical shape with a diameter of 78.4 ± 33.18 nm. The difference of nanomicelles size between DLS measurement compare to the size of nanomicelles in images of TEM is caused by hydrodynamic ratio role in the measured size of DLS method. Particle size blow than 200 nm is an ideal size for nanoparticular drug delivery systems. At physiological condition distribution of nanoparticles and drug conjugated macromolecules can be proportional to the blood content of tissues. At pathogenic condition of tumors, not only blood content of tissue is high but also permeability of endothelial barrier is higher than normal tissues and drainage of nanoparticles cannot happen by the lymphatic system. Therefore CPT-PAE-MPEG nanomicelles can be deposit into a tumor by enhanced permeation and retention (EPR) effect. On the other hand, low solubility and low blood protein binding of camptothecin cause its deposition in tissues immediately upon release and a long half-life in tumors.

##### The yield of drug conjugation and release analysis

The amount of conjugated CPT in CPT-PEA-MPEG micelles at ratio 60 : 40 was calculated using a UV-Vis spectrophotometer at 370 nm. The absorbance band of solutions was recorded, and the calibration curve of CPT was prepared. The average percentage of conjugated CPT in CPT-PEA-MPEG micelles base on the initial amount of CPT was 66.52% ± 0.06145.

**Fig. 5:**
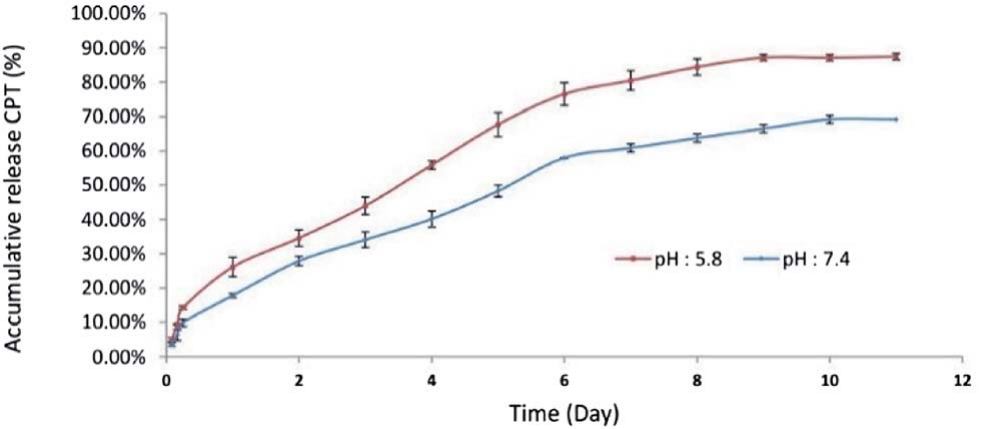
Cumulative release of CPT from CPT-PEA-MPEG micelles at pH 5.8 and pH 7, and 37 °C.

The release curve of CPT from CPT-PEA-MPEG micelles in 11 days is illustrated in Fig. 5. It showed that approximately 87 and 69% of CPT were released in 11 days at pH 5.8 and 7.4, respectively. In the first day, CPT-PEA-MPEG micelles released about 26.1 and 17.81% of CPT at pH 5.8 and 7.4, respectively. The release rate of CPT from CPT-PEA-MPEG micelles gradually increased in 6 days and reached to 76.51 and 58.01% at pH 5.8 and 7.4, respectively. These results indicated that CPT-PEA-MPEG micelles showed a distinguished pH-dependent drug release profiles. At pH 5.8, hydrolyze of ester bonds between drug and polymer is catalysed, and the release of CPT was faster. Therefore, acidic pH of the tumor can potentially trigger the rapid release of CPT at the tumor tissue.

##### *In vitro* cytotoxicity study

**Fig. 6:**
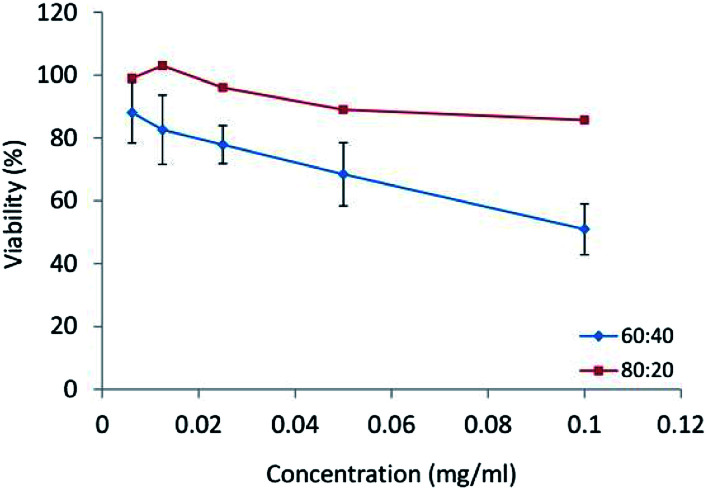
The cytotoxicity of nanomicelles at different ratios of MPEG : CPT (60 : 40 and 80 : 20) on HT-29 cell line.

In human tumor cell cytotoxicity assays, CPT and CPT derivatives demonstrated the ability for topoisomerase II inhibition and were highly cytotoxic to HT29 colon tumor cells.^32^ The HT29 cell line was incubated with different concentrations (0.1, 0.05, 0.025, 0.0125, 0.065 mg ml^−1^) of CPT-PAE-MPEG nanomicelles at 60 : 40 and 80 : 20 ratios of MPEG to CPT for 48 h and the cytotoxicity was assessed using MTT method (Fig. 6). The results revealed that nanomicelles at the ratio of 60 : 40 have higher cytotoxicity compared to nanomicelles at a ratio of 80 : 20. The IC_50_ was 0.1 mg ml^−1^ of nanomicelles for the ratio of 60 : 40. The greater cytotoxicity of 60 : 40 was attributed to its higher percentage of CPT. In the previous studies by other researchers, micelles of MPEG modified PAE as drug delivery systems have been used as a promising carrier to increase the efficacy of anti-cancer therapy with no inherent therapeutic effect.^33^ The novel PAE synthesized in this study not only shows an inherent cytotoxic effect but also can be potentially used as a carrier of other anti-cancer drugs. Clinical studies approved that combination therapy can reduce the risk of drug resistance and improved chemotherapy. Also, it had been proved that the concentration of polymer–drug conjugates in the tumor could reach 10–100 times higher than that for free drug.^34^ On the other hand, this polymeric micelle increases solubility, stability, and bioavailability of CPT. The cytotoxic activity of CPT depends on its structure. With conjugating CPT to PAE, the lactone ring can be stabilized. Camptothecin is an active anti-tumor drug, containing α-hydroxy-δ-lactone ring moiety. The intrinsic instability of CPT arises from the rapid hydrolysis of this ring in basic or neutral media, to give the open carboxylate form, which is essentially inactive. The reaction is reversible, and the lactone form predominates only at acidic pH. Pharmacokinetic studies have shown this pH-dependent hydrolytic equilibrium to be shifted toward the carboxylate form in plasma, in a species-dependent manner which the *in vivo* and *in vitro* biological activities of camptothecin are significantly greater for the lactone than those for the carboxylate form. Lactone form has a critical role in the passive diffusion of the drug into cancer cells as well as for successful interaction with the pharmacological target.^35^ It has been approved that the conjugation of CPT from its 20-OH group to a macromolecule, *e.g.*, PEG, poly(l-glutamic acid), starch and chitosan can enhance the solubility of the lactone ring and reduce the propensity for opening the ring.^12,36–38^

Regarding the obtained results, CPT is utilized as a constituent of nanomicelle caused inherent anti-cancer activity of micelles. On the other hand, prepared nanomicelles can be used as a proper carrier for other anti-cancer drugs. Co-delivery of two anticancer agents reduces the complexity of combination by enabling a better time, location, and dosage control and also results in synergistic effects.^39^ According to these reasons, we achieved an advantageous formulation which can be inherently used for cancer treatment and can act as a proper carrier for anti-cancer drugs.

## Conclusion

CPT is an active anti-tumor drug, effective on a wide range of tumors, *e.g.*, lung, breast, stomach, bladder, melanoma and ovary cancers. The therapeutic applications of CPT have been limited due to its instability at neutral pH, low solubility in aqueous medium and unwanted toxicity. In the present study, we designed a new PAE with high CPT conjugation efficiency and also, MPEG was used to enhance the stability and half-life of the carrier system.

The nanomicelles at two different ratios of MPEG : CPT (60 : 40 and 80 : 20) were prepared. The synthesis was confirmed with FT-IR and ^1^H NMR. Microemulsion method was employed to prepare the nanomicelles. The CMC, particle size analysis, surface charge, and transmission electron microscope imaging were performed. According to the results, for nanomicelles with a ratio of 60 : 40, the mean particle size was 118.9 ± 0.96 nm and the critical micelle concentration was 3.55 × 10^−5^ mg ml^−1^. *In vitro* cytotoxicity test was performed on HT29 cell line and the IC_50_ was achieved for 0.1 mg ml^−1^ of nanomicelles at MPEG : CPT ratio of 60 : 40. The results of this study showed that the CPT-PAE-MPEG nanomicelles at the ratio of 60 : 40 could be a proper choice to improve the physiochemical properties of CPT and enhance its therapeutic efficacy. Base of our knowledge it is the first time that camptothecin conjugated PAE-MPEG is synthesized and used for the preparation of nanomicelles with intrinsic anticancer effect and potential capacity to loading another anticancer drug with the purpose of dual drug delivery.

## Conflicts of interest

There are no conflicts to declare.

## Supplementary Material
